# Phytotoxic Activity and Structure–Activity Relationships of Radicinin Derivatives against the Invasive Weed Buffelgrass (*Cenchrus ciliaris*)

**DOI:** 10.3390/molecules24152793

**Published:** 2019-07-31

**Authors:** Marco Masi, Fabrizio Freda, Suzette Clement, Alessio Cimmino, Massimo Cristofaro, Susan Meyer, Antonio Evidente

**Affiliations:** 1 Dipartimento di Scienze Chimiche, Università di Napoli Federico II, Complesso Universitario Monte S. Angelo, Via Cintia 4, 80126 Napoli, Italy; 2 BBCA onlus, Via A. Signorelli 105, 00123 Rome, Italy; 3 Shrub Sciences Laboratory, U.S. Forest Service Rocky Mountain Research Station, 735 North 500 East, Provo, UT 84606, USA; 4 ENEA C.R. Casaccia, SSPT-BIOAG-PROBIO, Via Anguillarese 301, 00123 Rome, Italy

**Keywords:** buffelgrass, *Cochliobolus australiensis*, radicinin, derivatives, SAR, leaf puncture bioassay, phytotoxicity

## Abstract

Radicinin (**1**), is a fungal dihydropyranopyran-4,5-dione isolated together with some analogues, namely 3-*epi*-radicinin, radicinol, 3-*epi*-radicinol, and cochliotoxin (**2**–**5**), from the culture filtrates of the fungus *Cochliobolus australiensis*, a foliar pathogen of buffelgrass (*Cenchrus ciliaris*), an invasive weed in North America. Among the different metabolites **1** showed target-specific activity against the host plant and no toxicity on zebrafish embryos, promoting its potential use to develop a natural bioherbicide formulation to manage buffelgrass. These data and the peculiar structural feature of **1** suggested to carry out a structure-activity relationship study, preparing some key hemisynthetic derivatives and to test their phytotoxicity. In particular, *p*-bromobenzoyl, 5-azidopentanoyl, stearoyl, mesyl and acetyl esters of radicinin were semisynthesized as well as the monoacetyl ester of 3-*epi*-radicinin, the diacetyl esters of radicinol and its 3 epimer, and two hexa-hydro derivatives of radicinin. The spectroscopic characterization and the activity by leaf puncture bioassay against buffelgrass of all the derivatives is reported. Most of the compounds showed phytotoxicity but none of them had comparable or higher activity than radicinin. Thus, the presence of an α,β unsaturated carbonyl group at C-4, as well as, the presence of a free secondary hydroxyl group at C-3 and the stereochemistry of the same carbon proved to be the essential feature for activity.

## 1. Introduction

**Figure 1:**
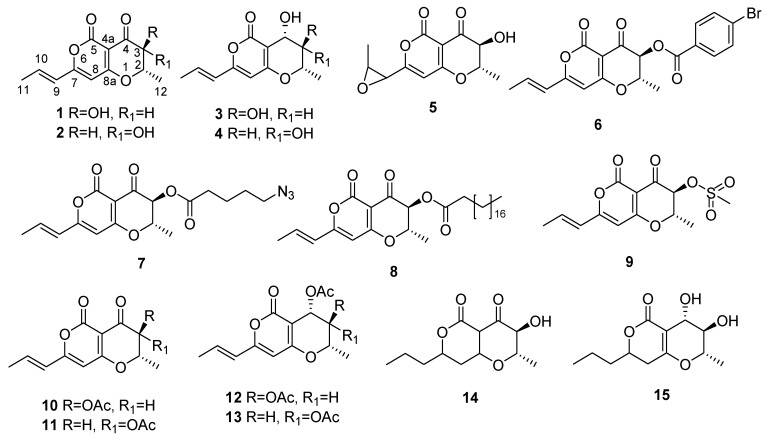
The structures of radicinin (**1**), 3-*epi*-radicinin (**2**), radicinol (**3**), 3-*epi*-radicinol (**4**), cochliotoxin (**5**), radicinin derivatives (**6**–**10**, **14**, and **15**), and the acetyl derivatives of 3-*epi*-radicinin, radicinol, and 3-*epi*-radicinol (**11**–**13**, respectively).

Buffelgrass (*Cenchrus ciliaris* or *Pennisetum ciliare*) is an important pasture grass in many semi-arid regions of the world but it is also an invasive weed in some areas of North America [1]. In the Sonoran Desert of southern Arizona it has infested thousands of acres of public and private lands, including Saguaro National Park and the Coronado and Tonto National Forests [2,3,4]. The increased fire frequency and intensity in the infested areas is negatively affecting the native species including the iconic saguaro cactus [5]. The main products currently used to manage buffelgrass are glyphosate and imazapyr. However, these broad-spectrum herbicides cause heavy damage to the non-target plants and have a negative environmental and ecological impact [6]. In the past decades the biological control has become an effective alternative to combat many weeds that invade natural systems [7,8]. In particular, the phytotoxins produced by weed pathogenic fungi are an efficient tool to design natural and safe bioherbicides [9]. Thus, *Cochliobolus australiensis* (recently classified as *Curvularia tsudae*) and *Pyricularia grisea*, two foliar pathogens that commonly occur on buffelgrass in the invaded North American range, were studied to evaluate their ability to produce phytotoxic metabolites that can potentially be used as natural herbicides against this weed. A total of 14 secondary metabolites belonging to different classes of natural compounds were purified from the in vitro cultures of these two pathogens [10,11,12]. When tested by leaf puncture assay on host plant at different concentrations, radicinin (**1**, Figure 1) and (10*S*,11*S*)-*epi*-pyriculol resulted to be the most promising compounds. Thus, their phytotoxic activity was also evaluated on non-host indigenous plants. Radicinin demonstrated high target-specific toxicity on buffelgrass, low toxicity to native plants and no teratogenic, sublethal, or lethal effects on zebrafish (*Brachydanio rerio*) embryos [13]. **1** is now under consideration for the development of target-specific bioherbicide to be used against buffelgrass, and a rapid and sensitive HPLC method for its quantification in complex mixtures was recently optimized in order to evaluate its production by different fungal strains and in different cultural conditions [14]. This manuscript reports the semisynthesis of some radicinin derivatives and the evaluation of their phytotoxic activity against buffelgrass. Furthermore, their phytotoxicity was also compared with that of the natural analogues 3-*epi*-radicinin, radicinol, 3-*epi*-radicinol, and cochliotoxin (**2**–**5**, Figure 1) and some of their derivatives (**6**–**15**, Figure 1) in order to obtain clues about the structure–activity relationship (SAR) of these compounds.

## 2. Results and Discussion

The buffelgrass pathogenic fungus *C. australiensis* was grown by fermentation and the natural compounds **1**–**5** were isolated from potato dextrose broth (PDB) cultures according to the procedures previously published [10,11]. The purity of **1**–**5** was >98%, as checked by ^1^H-NMR and LC–MS.

To investigate the structure activity relationship for radicinin as a target-specific phytotoxin against buffelgrass, seven derivatives (**6**–**10** and **14**–**15**, Figure 1) were prepared as reported in detail in the experimental part. The acetyl derivatives of 3-*epi*-radicinin, radicinol, and 3-*epi*-radicinol **11**–**13** were also prepared. All the derivatives were characterized as described in Materials and Methods in detail, and their ^1^H-NMR data are reported in Table 1 and Table 2.

Radicinin (**1**) by reaction with 4-bromobenzoyl chloride yielded its corresponding *p*-bromobenzoyl ester (**6**). Its ^1^H-NMR spectrum differed from that of **1** for the presence of the typical signals pattern of the aromatic *para*-disubstituted residue, appearing as two doublets at δ 7.94 and 7.65 (*J* = 8.6 Hz), and for the downfield shift (Δδ 1.52) at δ 5.52 of the signal of H-3. These data were very similar to those already reported by Robeson et al. [15]. As a stronger evidence of the derivatization, the ESI-MS spectrum showed the typical signals because of the presence of ^79^Br and ^81^Br isotopic peaks, at *m*/*z* 419 [M + H]^+^ and 421 [M + 2 + H]^+^, respectively.

Radicinin by esterification with 5-azidopentanoic acid was converted into the corresponding 5-azidopentanoyl derivative (**7**). Its ^1^H-NMR spectrum differed from that of radicinin for the downfield shift (Δδ = 1.25) of H-3 at δ 5.25 and for the presence of the signals pattern typical of 5-azidopentanoyl residue resonating as two triplets at δ 3.35 (*J* = 6.5 Hz) and 2.53 (*J* = 7.2 Hz) due to CH_2_-5′ and CH_2_-2′, and two multiplets at δ 1.80−1.72 and 1.68−1.71 due to CH_2_-3′ and CH_2_-4′. Further confirmation was obtained by the stretching of the –N_3_ bond in the IR spectrum, with a signal at 2097 cm*^−^*^1^ and by the ESI-MS spectrum, which showed both the dimeric sodiated [2M + Na]^+^ and protonated [M + H]^+^ forms at *m*/*z* 745 and 362, respectively.

**1** by reaction with stearoyl chloride afforded the corresponding acyl derivative (**8**). Its ^1^H-NMR spectrum, compared with that of radicinin, differed for the downfield shift (Δδ = 1.27) of H-3 at δ 5.27 and showed typical signals of the stearoil residue appearing as two triplets at δ 2.44 (*J* = 7.5 Hz) and 0.90 (*J* = 6.7 Hz) due to CH_2_-2′ and Me-18′ and the presence at δ 2.0–1.0 of the complex multiplet due to the protons of the residual 15 CH_2_ groups. The ESI-MS spectrum showed the protonated [M + H]^+^ form at *m*/*z* 517.

Radicinin by reaction with mesyl chloride in pyridine, afforded the corresponding mesyl ester (**9**). Its ^1^H-NMR spectrum, compared to that of **1** showed the downfield shift of H-3 (Δδ = 1.01) appearing as a doublet (*J* = 12.4 Hz) at δ 5.01 and the singlet of the mesyl group at δ 3.40. A further confirmation was obtained by the ESI-MS spectrum, which showed the dimeric sodiated [2M + Na]^+^ and the protonated [M + H]^+^ forms at *m*/*z* 651 and 315, respectively.

Radicinin (**1**) was acetylated by usual reaction with Ac_2_O and pyridine to yield the corresponding 3-*O*-acetylderivative (**10**). Its ^1^H-NMR differed from that of **1** essentially for the downfield shift of H-3 (Δδ = 1.28) appearing as singlet at δ 5.28 and the singlet of the acetyl group at δ 2.23. These data were very similar to those previously reported [16]. Furthermore, its ESI-MS spectrum showed the dimeric sodiated [2M + Na]^+^ and protonated [M + H]^+^ forms at *m*/*z* 579 and 279, respectively.

To investigate the role of the ethenyl side chain as well as those of the dihydropyranopyran-4,5-dione moiety, a catalytic hydrogenation of radicinin was carried out and two hexahydro derivatives (**14** and **15**) were obtained as main products. The ^1^H-NMR of both derivatives showed the absence of the olefinic pyron proton (H-8) and that (H-9) of the side chain at C-7. In fact ^1^H-NMR spectrum of **14** showed a triplet (*J* = 7.1 Hz) at δ 0.95 of the terminal methyl group (H_3_-11) of the propyl side chain at C-7, which in the COSY (Correlated spectroscopy) spectrum coupled with the adjacent methylene group (CH_2_-10) and this in turn with those of the other one (CH_2_-9) appearing as overlapped complex multiplet in the region of δ 1.62–1.43. The presence of the proton H-7 of the α-lactone ring appearing as dd (*J* = 12.2 and 4.7) at δ 4.42 was also significant, which in the COSY spectrum coupled with the broad double doublet (*J* = 13.2 and 4.7 Hz) and the double doublet (*J* = 13.2 and 12.2 Hz) at δ 12.19 and 1.63 of the proton of the adjacent methylene group (H_2_C-8). Another relevant difference was the upfield shift (Δδ 0.72) of the double quartet (*J* = 11.2 and 6.4 Hz) of H-2 resonating at δ 3.69 probably due to the saturation of the alpha α-pyrone ring that also generated the protons of the two headbridge carbons of the junction between the two rings appearing as multiplets at δ 4.33 and 1.62–1.43 (H-8a and H-4a, respectively). Furthermore, its ESI-MS spectrum showed the dimeric sodiated [2M + Na]^+^, the sodiated [M + Na]^+^ and protonated [M + H]^+^ forms at *m/z* 507, 265 and 243. Similarly, the ^1^H-NMR spectrum of **15** showed the signal of the propyl residue of C-7 with a triplet (*J* = 6.7 Hz) and three multiplets appearing at δ 0.96 (H_3_-11), 1.56 (H_2_-10), and 1.78 and 1.65 (H_2_-9). The proton assigned to H-7 resonated at *δ* 4.42 which in the COSY spectrum coupled with the two double doublets (*J* = 17.1 and 14.2 and *J* = 17.1 and 4.3 Hz) at δ 2.46 and 2.34 assigned to the proton of the adjacent methylene group of CH_2_-8. Moreover, the significant presence of the doublet (*J* = 9.2 Hz) of H-4, which coupled in the COSY spectrum with H-3, resonating as triplet (*J* = 9.2 Hz) δ 3.63 also coupled with H-2 appearing as a double quartet (*J* = 9.2 and 6.2 Hz) at δ 4.04. These coupling constants suggested the stereoselective reduction of the carbonyl group at C-4. H-4 appeared to be pseudoaxial located with respect to H-3. Furthermore, its ESI-MS spectrum showed the dimeric sodiated [2M + Na]^+^, the sodiated [M + Na]^+^ and protonated [M + H]^+^ forms at *m/z* 507, 265 and 243. The same sectrum also showed the ion at *m/z* 225 generated from the pseudomolecular ion by los of H_2_O.

3-*epi*-Radicinin (**2**) by reaction with Ac_2_O in pyridine, yielded the corresponding 3-*O*-acetyl derivative (**11**). Its ^1^H-NMR spectrum, differed from that of 3-*epi*-radicinin for the downfield shift of H-3 (Δδ = 1.0) resonating as a doublet (*J* = 3.8 Hz) at δ 5.51 and the singlet of the acetyl group at δ 2.19. Its ESI-MS spectrum showed both the protonated [M + H]^+^ and the dimeric sodiated [2M + Na]^+^ forms at *m*/*z* 279 and 579, respectively.

Radicinol (**3**) by acetylation, as above described, afforded the corresponding 3,4-*O*-*O*’-diacetyl derivative (**12**). Its ^1^H-NMR spectrum, compared with the spectrum of radicinol showed the typical downfield shifts of H-3 (Δδ = 1.40) and H-4 (Δδ = 1.0) appearing as two broad singlets at δ 5.12 and 5.78 respectively. The spectrum also showed the singlets of the overlapped signals of the two acetyl groups at δ 2.10. Its ESI-MS spectrum showed the protonated form [M + H]^+^ at *m*/*z* 323.

Similarly, 3-*epi*-radicinol was converted into its corresponding 3,4-*O*-*O*’-diacetyl derivative (**13**). The ^1^H-NMR spectrum of **13** differed from that of 3-*epi*-radicinol, for the downfield shifts of H-3 (Δδ = 1.31) and H-4 (Δδ = 1.00) appearing as a double doublet (*J* = 2.8 and 1.2 Hz) and a doublet (*J* = 2.8 Hz) at δ 5.16 and 5.72, respectively. The spectrum also showed the singlet at δ 2.11 due to the overlapped signals of the two acetyl groups. Its ESI-MS spectrum showed the protonated form [M + H]^+^ at *m*/*z* 323.

**Figure 2:**
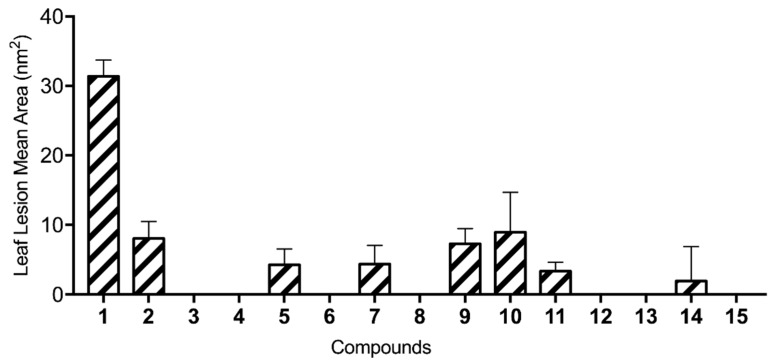
Results of the leaf puncture bioassay on buffelgrass (*Cenchrus ciliaris*) at a concentration of 2.5 × 10^−3^ M for the hemisynthetic derivatives (**6**–**15**) and the natural compounds (**1**–**5**).

The ten hemisynthetic derivatives (**6**–**15**) were tested by the buffelgrass leaf puncture assay at 2.5 × 10^−3^ M as reported in the Materials and Methods section in detail. Their activity was evaluated in comparison with that showed by the natural metabolites (**1**–**5**) previously reported by Masi et al. [13]. Overall, five derivatives (**6**, **8**, **12**, **13,** and **15**) produced no necrosis and were thus completely nontoxic to buffelgrass, while the other five compounds (**7**, **9**, **10**, **11**, and **14**) were moderately toxic (Figure 2).

A very important factor for the activity appear to be the carbonyl group of the dihydro *γ*-pyrone as the phytotoxicity was lost in **3**. This was also confirmed by the expected lacking of activity of radicinol diacetyl derivative (**12**). The stereochemistry at C-3 also plays a significant role to impart activity as was with the strong reduction observed by testing 3-*epi*-radicinin (**2**). This was confirmed by the very low activity of its acetyl derivative **11** and the total loss of activity observed by testing the diacetyl derivatives (**12** and **13**) of radicinol and 3-*epi*-radicinol. The hydrogenation of **1** generated **14** which showed the absence of α,β unsaturated carbonyl group explaining the strong loss of activity. The double bond of the side chain at C-7 also plays a role to impart activity as demonstrated by the reduction of phytotoxicity observed by testing **5** and confirmed by the decrease of the activity of **14.** The acyl derivatives of **1** showed a strong or total loss of activity, which was only in part retained by the acetyl and the mesyl derivatives (**9** and **10**). These compounds are probably more easily hydrolysable in comparison to the other compounds (**6**, **7,** and **8**) as their conjugated bases are more stabilized for resonance.

**Figure 3:**
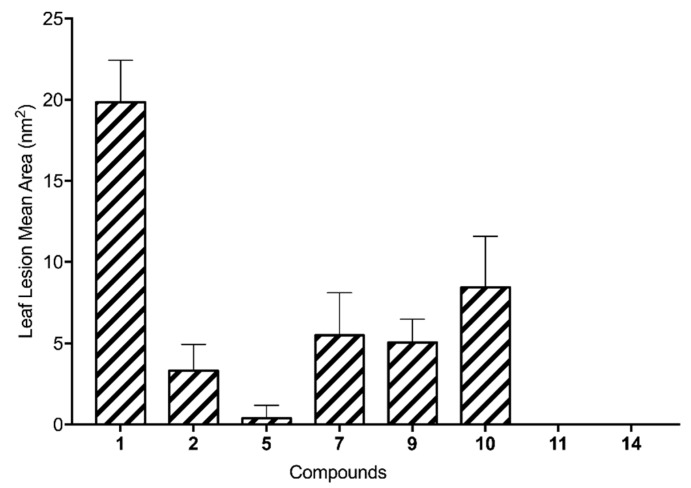
Results of the leaf puncture bioassay on buffelgrass (*Cenchrus ciliaris*) at a concentration of 10^−3^ M for the hemisynthetic derivatives (**7**, **9**, **10**, **11**, and **14**) and the natural compounds (**1**, **2,** and **5**).

The five hemisynthetic derivatives (**7**, **9**, **10**, **11**, and **14**) that showed toxic activity on buffelgrass leaves at 2.5 × 10^−3^ M were also tested at a lower concentration of 10^−3^ M (Figure 3), confirming the results obtained at higher concentration.

## 3. Materials and Methods

### 3.1. General Experimental Procedures

IR spectra were recorded as deposit glass film on a Perkin Elmer Thermo Nicolet 5700 FT-IR spectrometer (Thermo Scientific, Waltham, MA, USA). UV spectra were measured in MeOH on a Jasco V-530 spectrophotometer (Jasco, Tokyo, Japan). ^1^H-NMR spectra were recorded at 400 or 500 MHz in CDCl_3_ on Bruker (Bruker, Karlsruhe, Germany) and Varian (Varian, Palo Alto, CA, USA) instruments. ESIMS (Electrospray ionization mass spectrometry) were recorded using LC/MS ESIMS-TOF (Electrospray ionization mass spectrometry –Time of flight) system (Agilent 6230B, HPLC 1260 Infinity) (Agilent Technologies, Milan, Italy). Analytical, preparative and reverse phase TLCs (Thin layer chromatography) were carried out on silica gel (Kieselgel 60, F_254_, 0.25, 0.5 mm, and RP-18 F_254_s respectively) plates (Merck, Darmstadt, Germany). The spots were visualized by exposure to UV radiation, or by spraying first with 10% H_2_SO_4_ in MeOH, and then with 5% phosphomolybdic acid in EtOH, followed by heating at 110 °C for 10 min. Column chromatography was performed using silica gel (Merck, Kieselgel 60, 0.063–0.200 mm).

### 3.2. Fungal Strains

*Cochliobolus australiensis* (LJ-4B) strains used in this study were isolated from diseased buffelgrass tissue collected in Saguaro National Monument, Arizona, AZ, USA, in autumn 2014 and near La Joya, Hidalgo County in south Texas, USA, in September 2014, respectively.

### 3.3. Isolation of Fungal Metabolites

The fungal metabolites **1**–**5** were isolated from in vitro PDB (potato dextrose broth) cultures of *C. australiensis* according to procedures previously reported [10,11].

### 3.4. Preparation of Semisynthetic Derivatives

#### 3.4.1. *p*-Bromobenzoyl Ester of Radicinin (**6**)

To radicinin (**1**, 5.0 mg), dissolved in anhydrous MeCN (300 µL), DMAP (5.0 mg) and *p*-bromobenzoylchloride (5.0 mg) were added. The reaction mixture was left under stirring for 5 h. It was then quenched with a 1 N NaHCO_3_ and extracted with CH_2_Cl_2_. The residue obtained by evaporation was then purified by preparative TLC eluted with CHCl_3_:*i*-PrOH (9:1) affording the *p*-bromobenzoyl ester of radicinin (**6**, 4.17 mg). Derivative **6** had: IR *ν*_max_ 1763, 1730, 1602, 1532, 1433 cm^−1^; UV λ_max_ nm (log ε) 345 (4.23), 247 (4.40), 224 (4.23). These data were very similar to those already reported by Robeson et al. [15]; ^1^H-NMR, see Table 1; MS (ESI-TOFMS) *m*/*z* 421 [M + 2 + H]^+^, 419 [M + H]^+^.

#### 3.4.2. 5-Azidopentanoyl Ester of Radicinin (**7**)

DCC (5.6 mg) and 5-azidopentanoic acid (20 µL) were added to a solution of radicinin (**1**) in pyridine (5.0 mg in 300 µL). The mixture was kept under stirring at room temperature for 4 days and then dried under N_2_ stream by evaporation of the azeotope obtained by adding MeOH and C_6_H_6_. The residual oil was then purified by TLC eluted with CHCl_3_-*i*-PrOH (97:3) affording 5-azidopentanoyl ester of radicinin (**7**, 4.38 mg). Derivative **7** had: IR ν_max_ 2097, 1754, 1601, 1523, 1433 cm^−1^; UV λ_max_ nm (log ε) 341 (4.15), 221 (4.14); ^1^H-NMR, see Table 1; MS (ESI-TOFMS) *m*/*z* 745 [2M + Na]^+^, 362 [M + H]^+^.

#### 3.4.3. Stearoyl Ester of Radicinin (**8**)

Stearoyl chloride (12.8 mg), DMAP (1.6 mg), and DCC (9.0 mg) were added to radicinin (**1**, 5.0 mg) in CH_2_Cl_2_ (300 µL) together with 9 mg of DCC. The reaction mixture was kept under stirring for 3 days and was then dried under N_2_ stream. The residual oil was purified by preparative TLC eluted with *n*-hexane-EtOAc (55:45) yielding the stearoyl ester of radicinin (**8**, 1.81 mg). Derivative **8** had: IR ν_max_ 1750, 1532, 1433 cm^−1^; UV λ_max_ nm (log ε) 341 (4.15), 221 (4.14); ^1^H-NMR, see Table 1; MS (ESI-TOFMS) *m*/*z* 517 [M + H]^+^.

#### 3.4.4. Mesyl Ester of Radicinin (**9**)

Mesyl chloride (23 µL) was added to radicinin (**1**, 5.0 mg) dissolved in CH_2_Cl_2_ (300 µL) and pyridine (40 µL). The reaction mixture was kept overnight and then quenched with a 1 N solution of NaHCO_3_. The mixture was then extracted with EtOAc and the organic extract purified by TLC eluted with CHCl_3_-*i*-PrOH (97:3) affording the mesyl ester derivative of radicinin (**9**, 3.34 mg). Derivative **9** had: IR ν_max_ 1757, 1602, 1532, 1434 cm^−1^; UV λ_max_ nm (log ε) 342 (4.10), 224 (4.12); ^1^H-NMR, see Table 1; MS (ESI-TOFMS) *m*/*z* 651 [2M + Na]^+^, 315 [M + H]^+^.

#### 3.4.5. Acetyl Ester of Radicinin (**10**)

Radicinin (**1**, 4 mg), dissolved in pyridine (100 µL), was acetylated with Ac_2_O (5 µL) to obtain derivative **10**. The reaction was carried out under stirring for 3 h at room temperature. It was stopped with MeOH, and the azeotrope formed by addition of C_6_H_6_ was evaporated under nitrogen stream. The residue was then purified by TLC eluted with CHCl_3_-*i*-PrOH (95:5) yielding **10** (2.3 mg) as an amorphous solid. Derivative **10** had: IR ν_max_ 1754, 1602, 1531, 1433 cm^−1^; UV λ_max_ nm (log ε) 345 (4.12), 227 (4.15); ^1^H-NMR data (see Table 1) were very similar to those previously reported by Aldrich et al. [16]; MS (ESI-TOFMS) *m*/*z* 579 [2M + Na]^+^, 279 [M + H]^+^.

#### 3.4.6. Acetyl Ester of 3-*epi*-Radicinin (**11**)

3-*epi*-radicinin (**2**, 3 mg), dissolved in pyridine (100 µL), was acetylated with Ac_2_O (3 µL). The reaction was carried out following the same procedure used for conversion of **1** into **10** yielding the corresponding acetyl ester of 3-*epi*-radicinin (**11**, 1.76 mg). Derivative **11** had: IR ν_max_ 1750, 1602, 1532, 1433 cm^−1^; UV λ_max_ nm (log ε) 348 (4.10), 228 (4.12); ^1^H-NMR, see Table 2; MS (ESI-TOFMS) *m*/*z* 579 [2M + Na]^+^, 279 [M + H]^+^.

#### 3.4.7. Diacetyl Ester of Radicinol (**12**)

To radicinol (**3**, 5 mg), dissolved in pyridine (100 µL), Ac_2_O (10 µL) was added. The reaction was kept under stirring at room temperature for 4 h and was then dried as previously reported for the preparation of **10**. The residue was then purified by TLC eluted with CHCl_3_-*i-*PrOH (98:2) to afford the derivative **12** (3.46 mg). Derivative **12** had: IR ν_max_ 1680, 1650, 1610, 1560 cm^−1^; UV λ_max_ nm (log ε) 319 (4.04), 271 (3.40), 261 (3.38) 225 (4.51); ^1^H-NMR, see Table 2; MS (ESI-TOFMS) *m*/*z* 323 [M + H]^+^.

#### 3.4.8. Diacetyl Ester of 3-*epi*-Radicinol (**13**)

To *epi*-radicinol (**4**, 5 mg), dissolved in pyridine (100 µL), Ac_2_O (10 µL) was added. The reaction was carried out following the same procedure used for the conversion of **2** into **11** yielding the corresponding acetyl ester of 3-*epi*-radicinol (**13**, 4.36 mg). Derivative **13** had: IR ν_max_ 1682, 1649, 1611, 1560 cm^−1^; UV λ_max_ nm (log ε) 318 (4.05), 271 (3.41), 260 (3.36) 225 (4.50); ^1^H-NMR, see Table 2; MS (ESI-TOFMS) *m*/*z* 323 [M + H]^+^.

#### 3.4.9. Hydrogenation of Radicinin: Derivatives **14** and **15**

Radicinin (**1**, 6 mg), dissolved in MeOH (1 mL), was added to a presaturated suspension of 10% Pd/C in MeOH (1 mL). Hydrogenation was carried out at room temperature and atmospheric pressure with continuous stirring. After 3h, the reaction was stopped by filtration on short silica gel column, eluted with CHCl_3_-*i-*PrOH (9:1) and the clear solution was evaporated under reduced pressure. The crude residue was purified by preparative TLC eluted with CHCl_3_-*i-*PrOH (95:5) affording two different hydrogenated derivatives as main products (**14** and **15**, 2.12 and 1.40 mg respectively). Derivative **14** had: IR ν_max_ 3452, 1726, 1673, 1212 cm^−1^; UV λ_max_ nm (log ε) <220 nm; ^1^H-NMR, see Table 2; MS (ESI-TOFMS) *m*/*z* 507 [2M + Na]^+^, 265 [M + Na]^+^ and 243 [M + H]^+^. Derivative **15** had: IR ν_max_ 3450, 1681, 1643 cm^−1^; UV λ_max_ nm (log ε) <220 nm; ^1^H-NMR, see Table 2; MS (ESI-TOFMS) *m*/*z* 507 [2M + Na]^+^, 265 [M + Na]^+^, 243 [M + H]^+^ and 225 [M − H_2_O + H]^+^.

### 3.5. Leaf Puncture Bioassays

The ten hemisynthetic derivatives (**6**–**15**) were first assayed at 2.5 × 10^−3^ M for phytotoxicity on the leaves of buffelgrass (*Cenchrus ciliaris*) and those active at this concentration were then bioassayed at 10^−3^ M. Compounds were first dissolved in MeOH (final concentration 4%) and stock solutions at the two concentrations using sterile distilled water were then prepared. An incision of ca. 3 mm was made on the adaxial surface of each leaf section of 3 cm with an insulin needle. The leaf sections were placed in groups of six on the surface of a water-saturated filter paper in each of the four petri dishes. A total of five leaf sections in each petri dish were tested with the solution containing the compound, while one leaf section was used as a negative control (4% MeOH only). A droplet (10 μL) of the appropriate solution was applied over each needle incision using a micropipette. The dishes were sealed with parafilm and incubated at 24 °C for 3 days in a temperature-regulated chamber under a photoperiod of 14–10 h (light/dark). After 3 days of treatment, necrotic lesion development was evaluated by removing the petri dish cover, placing a glass disc on the leaf sections to flatten them into a single plane, and photographing each dish with its leaf sections. Each acquired image was then analyzed with the software ImageJ to measure the necrotic area caused by the solution.

### 3.6. Statistical Analyses

Statistical analyses were carried out using the GraphPad Prism 8 software (GraphPadSoftware, San Diego, CA, USA). Data were represented as the mean ± standard deviation and analyzed for statistical significance using ordinary one-way or two-ways analysis of variance and multiple comparisons. For all test, *p <* 0.5 was considered to indicate a statistically significant difference.

## 4. Conclusions

These results obtained in this study demonstrated that the α,β unsaturated carbonyl group at C-4 and the stereochemistry at C-3 are important structural features to impart phytotoxicity. Furthermore, the unsaturation of propenyl side chain also play a role to impart activity.

Thus, radicinin appears to be the most active compound suitable to develop a target-specific bioherbicide for buffelgrass control. Considering the very low production of this compound by different strains of the fungus *C. australiensis* [14] and the difficulties to scale-up the cultures via a fermenter, a suitable alternative appears to be its total enantioselective synthesis.

## Figures and Tables

**Table 1:** ^1^H-NMR data of radicinin (**1**) and some of its derivatives (**6**–**10**) *^a^*.

Position	1	6	7 *^b^*	8 *^c^*	9	10
2	4.41 dq (12.4, 6.3)	4.91 dq (10.3, 6.3)	4.73 m	4.74 dq (12.4, 6.1)	4.72 dq (12.4, 6.0)	4.74 dq (11.0, 6.4)
3	4.00 d (12.4)	5.52 d (10.9)	5.25 d (11.0)	5.27 d (12.4)	5.01 d (12.4)	5.28 d (11.0)
8	5.94 s	5.90 s	5.84 s	5.87 s	5.90 s	5.87 s
9	6.12 d (15.5)	6.10 d (15.5)	6.05 d (15.5)	6.07 d (15.5)	6.09 d (15.5)	6.07 d (15.5)
10	7.00 sext. (7.1)	6.99 sext. (7.1)	6.97 sext. (7.1)	6.99 sext. (7.1)	7.05 sext. (7.1)	6.99 sext. (7.1)
11	2.01 d (7.1)	1.99 d (7.1)	1.95 d (7.1)	1.98 d (7.1)	2.01 d (7.1)	1.99 d (7.1)
Me	1.60 d (6.3)	1.61 d (6.3)	1.54 d (6.3)	1.56 d (6.1)	1.69 d (6.0)	1.57 d (6.4)
CO-Me						2.23 s
2′-6′		7.94 d (8.6)				
3′-5′		7.65 d (8.6)				
S-Me					3.40 s	

*^a^* The chemical shifts are in δ values (ppm) from TMS. *^b^* The signals of the acyl group of this compound are at δ: 3.35 (t, *J* = 6.5 Hz, CH_2_-5′), 2.53 (t, *J* = 7.2 Hz, CH_2_-2′), 1.80–1.72 (m, CH_2_-3′), 1.68–1.71 (m, CH_2_-4′). *^c^* The signals of the acyl group of this compound are at δ: 2.44 (t, *J* = 7.5 Hz, CH_2_-2′), 2.0–1.0 (m, CH_2_-3′-CH_2_-17′), 0.90 (t, *J* = 6.7 Hz, Me-18′).

**Table 2:** ^1^H-NMR data of acetyl derivatives of 3-*epi*-radicinin, radicinol and 3-*epi* radicinol (**11**–**13**) and of radicinin derivatives (**14** and **15**) *^a^*.

Position	11	12	13	14	15
2	4.90 dq (3.8, 6.4)	4.58 br. s	4.43 dd (6.9,1.2)	3.69 dq (11.2, 6.4)	4.04 dq (9.2, 6.2)
3	5.51 d (3.8)	5.78 br. s	5.16 dd (2.8, 1.2)	4.04 d (11.2)	3.63 t (9.2)
4		5.12 br. s	5.72 d (2.8)		4.49 d (9.2)
4a				1.62–1.43 *^c^*	
7				4.46 dd (12.2, 4.7)	4.42 m
8	5.88 s	5.81 s	5.79 s	2.19 br dd (13.2, 4.7) 1.63 dd (13.2, 12.2)	2.46 dd (17.1, 14.2) 2.34 dd (17.1, 4.3)
8a				4.33 m	
9	6.07 d (15.5)	5.98 d (15.5)	6.02 dd (15.5, 1.4)	1.62–1.43 (2H) *^c^*	1.78 m 1.65 m
10	6.98 sext (7.1)	6.77 sext (7.1)	6.78 sext (7.1)	1.62–1.43 (2H) *^c^*	1.56 m (2H)
11	1.98 d (7.1)	1.98 d (7.1)	1.94 d (7.1)	0.95 t (7.1)	0.96 t (6.7)
Me	1.47 d (6.4)	1.47 d (6.3)	1.39 d (6.3)	1.41 d (6.4)	1.57 d (6.2)
CO-Me	2.19 s	2.10 s *^b^*	2.11 s *^b^*		

*^a^* The chemical shifts are in δ values (ppm) from TMS. *^b^* This is the signal of two overlapped acetyl groups. *^c^* Overlapped signals.
